# Nontuberculous Mycobacterial Pulmonary Disease in an Immunocompromised Patient

**DOI:** 10.7759/cureus.6065

**Published:** 2019-11-03

**Authors:** Ranjan K Singh, Dipak Kumar

**Affiliations:** 1 Internal Medicine, Anti-Retroviral Therapy Centre, District Hospital, Khagaria, IND

**Keywords:** hiv, ntm, mai

## Abstract

In this article, we discuss the nontuberculous mycobacterial pulmonary disease in a 40-year-old HIV-seropositive female patient. The patient has a history of pulmonary tuberculosis, experienced two years ago. At the time, she was treated successfully with anti-tuberculous therapy. A chest x-ray (CXR) and computed tomography (CT) scan of the chest showed a thin-walled cavitary lesion in the right lung. In addition, the tree-in-bud sign, indicative of airway obstruction, was present on CT imaging. Fluorescence microscopy using auramine staining showed acid-fast bacilli (AFB) in sputum smears on more than two samples. Mycobacterium tuberculosis was not detected in the nucleic acid amplification test in the same sample. The AFB identified were mycobacteria other than tubercle bacilli, i.e., nontuberculous mycobacteria, that cause cavitary lung disease. Culture in liquid media and subsequent molecular analysis showed Mycobacterium avium complex (MAC). The patient is now being treated with a multidrug regimen of antibiotics and has improved, with documented sputum conversion.

## Introduction

Nontuberculous mycobacteria (NTM) are a group of bacilli, other than tuberculous, containing more than 160 species. Of these species, Mycobacterium avium complex (MAC), M. kansasii, M. abscessus, and M. xenopi frequently cause pulmonary disease in patients of compromised immune status. 

NTM are ubiquitous in the soil and water. In contrast to M. tuberculosis, NTM is not transmitted through person-to-person contact but, rather, spread from soil and water through aerosolized droplets. The prevalence of nontuberculous mycobacteria pulmonary disease (NTM-PD) varies from one region to another; for example, there are 5.9 cases per 100,000 population in Spain, 6.5 cases per 100,000 population in the United Kingdom (UK), and 1.3 cases per 100,000 population in France [1]. The disease happens to be a frequently missed diagnosis because the clinical features of NTM-PD are indistinguishable from that of pulmonary tuberculosis. Also, sputum smear microscopy does not distinguish NTM from M. tuberculosis, as bacilli in both cases are acid-fast and morphologically similar in appearance. Adhikaram found that patients with NTM-PD have been erroneously diagnosed as having tuberculosis and multidrug-resistant tuberculosis in 4.5 to 15% and 18 to 20% cases, respectively [2]. Guidelines for detecting NTM-PD were established in 2007 by the American Thoracic Society and the Infectious Diseases Society of America [3]. The guidelines include details of clinical presentation, such as fever, cough, hemoptysis, and breathlessness, along with radiological features, such as cavitary lesions, bronchiectatic changes, and nodules. Pleural effusion and hilar lymphadenopathy are uncommon in NTM. Computed tomography (CT) of the chest shows a thin-walled cavity, multiple nodules, and the tree-in-bud sign. Sputum analysis from samples obtained on two occasions or samples from bronchoalveolar lavage on one occasion should show NTM.

## Case presentation

**Figure 1:**
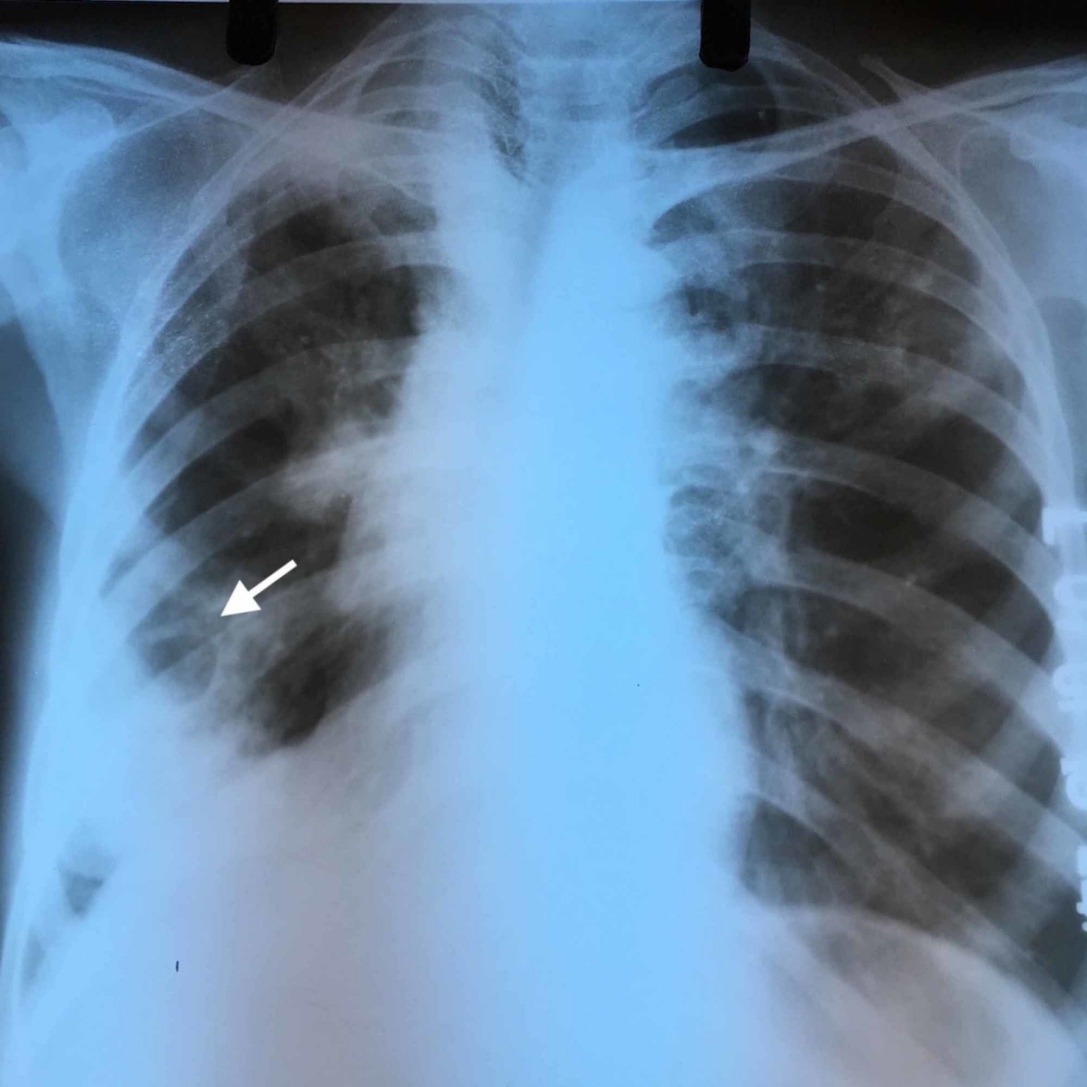
Chest X-ray showing a cavitary lesion in the lower zone of the right lung field

A 40-year-old human immunodeficiency virus (HIV)-seropositive female was complaining of fever, cough, and hemoptysis for two months. She had been taking antiretroviral therapy (tenofovir, lamivudine, and efavirenz) for the past seven years. Her recent CD4 cell count and viral load were 476/mm^3^ and 1442 copies/ml respectively. She had pulmonary tuberculosis (drug-sensitive Mycobacterium tuberculosis) two years ago and responded completely to nine months of anti-tuberculous treatment. A recent clinical examination revealed diffuse rhonchi on the right side of her chest. Chest x-ray (CXR) showed a cavitary lesion in the right lung field with homogenous opacity in the middle zone of her right lung (Figure 1).

**Figure 2:**
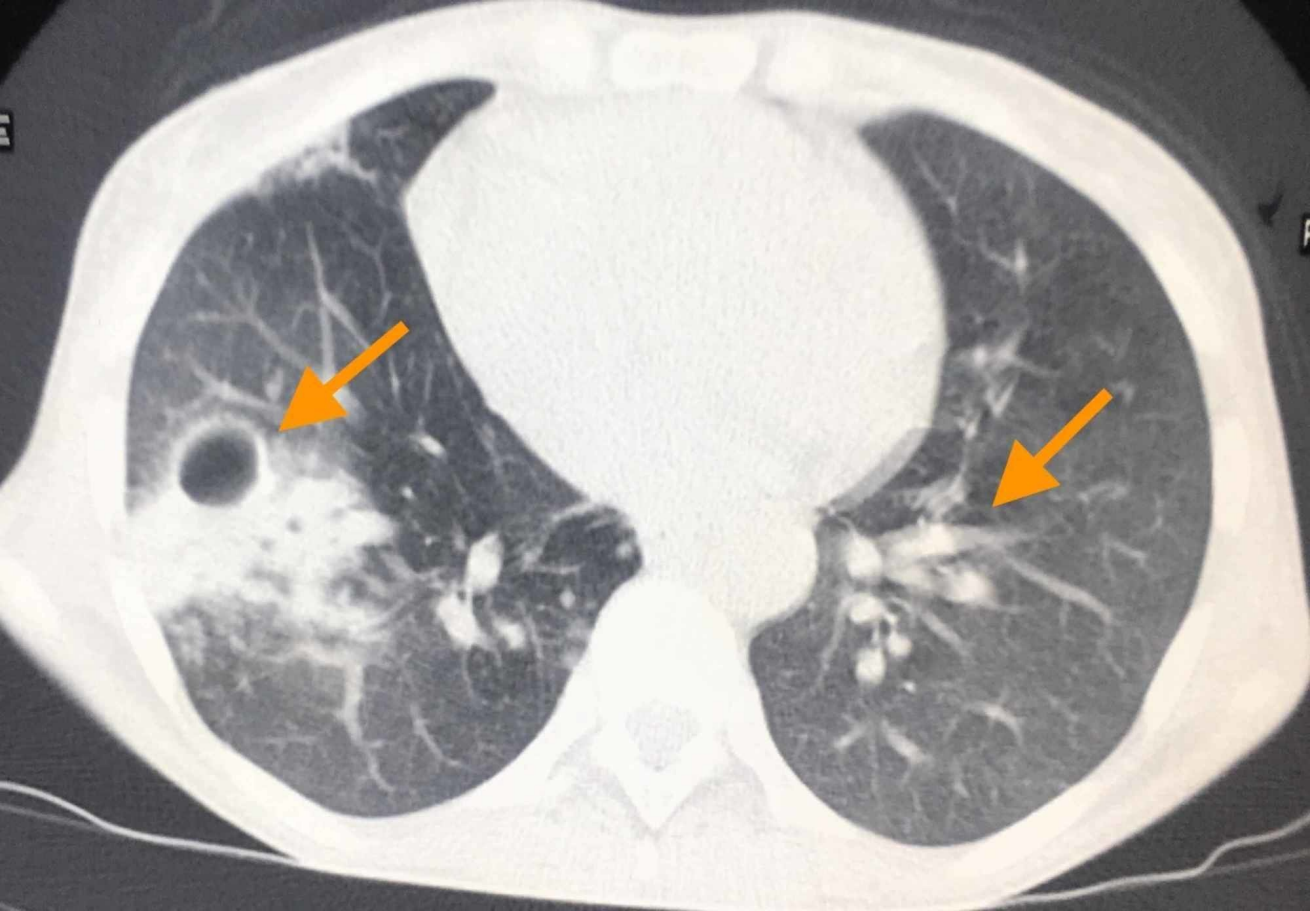
CT scan showing cavitary lesion in the right lung and tree-in-bud sign in the left lung

A CT scan of her chest showed a cavitary lesion in the right middle lung and a tree-in-bud appearance in the left lung (lingual) (Figure 2).

**Figure 3:**
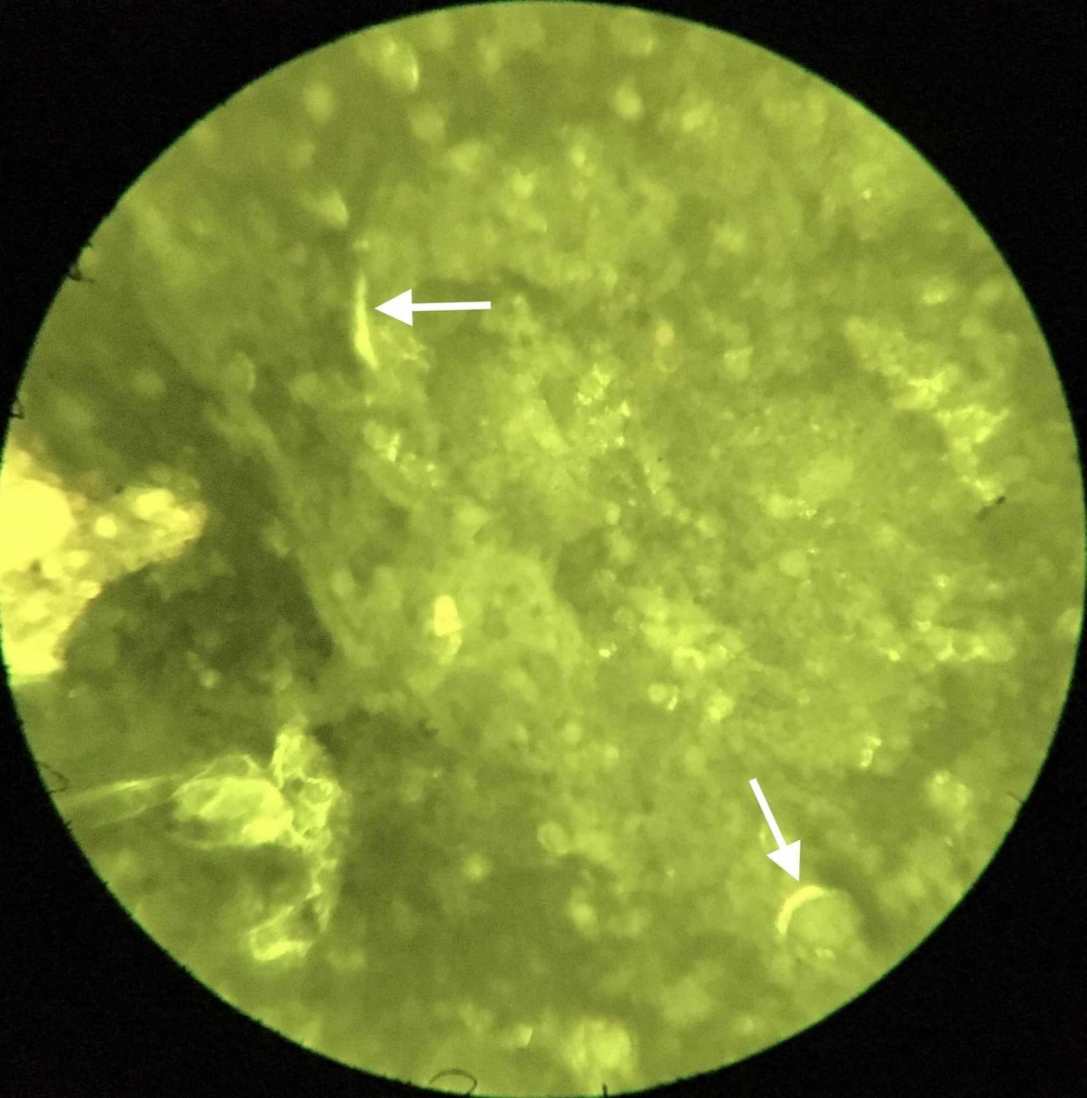
Sputum smear with auramine staining under fluorescence microscopy (magnification 200X) showing acid-fast bacilli

Sputum collected in the morning was subjected to fluorescence microscopy after auramine staining and nucleic acid amplification test (NAAT) for M. tuberculosis. Microscopy showed acid-fast bacilli (AFB) (Figure 3) but the NAAT was negative for M. tuberculosis.

Again, sputum was collected on the second day and examined, with similar findings as for the previous sputum collection. Culture on liquid media showed NTM, which were sent to the National Reference Laboratory for mycobacterial diseases in Delhi, for species identification and determination of sensitivity to drugs. The species was identified as MAC. 

The patient was treated with azithromycin 500 mg once daily (OD), rifampicin 450mg OD, ethambutol 600mg OD, and amikacin (Mikacin) 750mg intravenously (IV) three times a week for two months, at which point in time the Mikacin injection was stopped. Sputum conversion was detected after four months and treatment continued for a further 12 months after sputum conversion.

## Discussion

Like solid organ transplant recipient patients, HIV-seropositive patients have an increased risk for infection with NTM because of depressed cell-mediated immunity. Several preexisting lung diseases such as cystic fibrosis, bronchiectasis, prior pulmonary tuberculosis, chronic obstructive pulmonary disease, and pneumoconiosis predispose to NTM. HIV infection and prior pulmonary tuberculosis led to the acquisition of NTM-PD in this case. 

Possible differential diagnoses for a thin-walled cavitary pulmonary lesion in HIV-seropositive cases include tuberculosis, invasive aspergillosis, Pneumocystis jirovecii pneumonia, endemic fungal infections, rheumatoid arthritis, as well as nontuberculous mycobacterium. CT images show a cavitary lesion in 46%, nodules in 29%, and the tree-in-bud sign in 21% of cases of NTM-PD [4]. In our case, a thin built middle age lady has tree-in-bud in lingula and cavitary lesion in the right middle lobe on CT imaging. A similar pattern of tree-in-bud sign localized in right middle lobe or lingula in an immunocompetent middle age lady has been described as “lady Windermere syndrome” 25 years back. The tree-in-bud sign is not pathognomonic to NTM-pulmonary disease because this sign can be found in infective bronchiolitis, cystic fibrosis, endobronchial tuberculosis, allergic bronchopulmonary aspergillosis, and rheumatoid arthritis [5]. 

Microscopic detection of AFB in sputum smear and a simultaneous negative NAAT for M. tuberculosis strongly indicate NTM-PD [6]. However, a culture of the sputum specimen is still regarded as the gold standard method for microbiological confirmation.

Dissemination of NTM infection depends on CD4 T cell status i.e. < 50/mm^3^ in HIV patients. Prompt initiation of antiretroviral drugs with the maintenance of CD4 T cells >100/mm^3^ prevents dissemination. NTM infections were observed among patients who have viral load >1000 copies/ml, however, there is statistically no difference in the incidence of NTM infections among different baseline viral loads [7]. 

## Conclusions

The prevalence of NTM-PD varies geographically, and the disease is easily missed, commonly diagnosed instead as pulmonary tuberculosis and drug-resistant pulmonary tuberculosis. Fluorescence microscopy of sputum smear and simultaneous NAAT (of the same sample) provide innovation in the diagnosis of NTM-PD. A culture of sputum for species identification and determining drug sensitivity is regarded as the gold standard method for confirmation of the diagnosis of NTM. NTM dissemination depends on the lower CD4 T cell status of the patient.
